# Exploring the Link between Inflammatory Biomarkers and Head and Neck Cancer: Understanding the Impact of Smoking as a Cancer-Predisposing Factor

**DOI:** 10.3390/biomedicines12040748

**Published:** 2024-03-27

**Authors:** Jarosław Nuszkiewicz, Joanna Wróblewska, Marlena Budek, Jolanta Czuczejko, Alina Woźniak, Marta Maruszak-Parda, Karolina Szewczyk-Golec

**Affiliations:** 1 Department of Medical Biology and Biochemistry, Faculty of Medicine, Ludwik Rydygier Collegium Medicum in Bydgoszcz, Nicolaus Copernicus University in Toruń, 24 Karłowicza St., 85-092 Bydgoszcz, Poland; joanna.wroblewska@cm.umk.pl (J.W.); mmarkiewicz@doktorant.umk.pl (M.B.); al1103@cm.umk.pl (A.W.); karosz@cm.umk.pl (K.S.-G.); 2 Department of Psychiatry, Faculty of Medicine, Ludwik Rydygier Collegium Medicum in Bydgoszcz, Nicolaus Copernicus University in Toruń, 9 M. Curie Skłodowskiej St., 85-094 Bydgoszcz, Poland; joczu@cm.umk.pl; 3 Department of Nuclear Medicine, Oncology Centre Prof. Franciszek Łukaszczyk Memorial Hospital, Bydgoszcz, 2 Dr I. Romanowskiej St., 85-796 Bydgoszcz, Poland; m.maruszak@gmail.com

**Keywords:** biomarkers, cytokines, head and neck cancer, inflammation, risk factors, smoking

## Abstract

Head and neck cancer (HNC) is associated with significant morbidity globally, with smoking recognized as a key risk factor. This study investigates the interplay between smoking and inflammatory biomarkers in HNC development. The study involved 50 HNC patients, divided into smoking and non-smoking groups, and a control group of 30 healthy individuals. Serum levels of 48 cytokines, chemokines, growth factors, and other inflammatory markers were meticulously assessed. Significant differences in the levels of an extensive panel of inflammatory markers were observed between the patient groups and healthy controls. Elevated macrophage colony-stimulating factor (M-CSF) in both HNC groups implicated increased activity in pathways known for immunomodulation, proliferation, and angiogenesis during HNC cancerogenesis. In contrast, non-smokers with HNC demonstrated higher levels of interleukin 10 (IL-10) and interleukin 15 (IL-15), suggesting a more robust immune response. Platelet-derived growth factor BB (PDGF-BB) levels were particularly high in smokers with HNC. Smoking seems to alter the levels of crucial biomarkers in HNC, potentially affecting disease progression and responses to treatment. The data indicate that smokers may experience a more aggressive cancer phenotype, while non-smokers maintain a profile suggestive of a more active and effective immune response against HNC.

## 1. Introduction

Cancer ranks among the primary global causes of mortality [1]. An estimation suggests that, in 2012, there were 14.1 million new cases of cancer and 8.2 million cancer-related deaths worldwide [2]. Head and neck cancers (HNCs) are a relatively uncommon group of neoplasms but represent a significant clinical and social problem. Annually, it is estimated that more than 500,000 people worldwide are diagnosed with HNC [3]. According to the latest available data, in 2020, there were 151,000 new cases of HNCs in Europe [4]. Despite significant advancements in diagnostic and therapeutic methods, HNCs are still associated with unfavorable prognoses. HNCs affect various organs located in the head and neck region, including the lip, oral cavity, tongue, gums, pharynx, tonsils, larynx, paranasal sinuses, and salivary glands [5]. The symptoms of HNC may vary depending on the location of the cancer at the initial stage. Common symptoms include pain and the ulceration of the affected tissue, which may gradually lead to breathing, swallowing, and speech difficulties [6]. Surgery, chemotherapy, and radiotherapy are the most commonly used forms of treatment for patients with HNC, and these methods are typically used in combination therapy [7].

Numerous risk factors that increase the probability of developing HNC have been identified [8,9]. Carcinogenesis may be caused by irritation of the oral cavity and throat mucous membranes by cigarette smoke or hard alcohol, as well as chronic mechanical damage to the tissues due to ill-fitting dentures or broken teeth [10]. Around 120 million people, nearly 28% of adults in the European Union, are estimated to be smokers and approximately 650,000 people die annually due to smoking [11,12]. Smoking has not only been linked to a high risk of HNCs but also to lung cancer, coronary artery disease, Alzheimer’s disease, stroke, and decreased bone density [13,14]. Tobacco smoke consists of over 5000 chemicals, many of which are toxic and carcinogenic [15]. These toxins can directly or indirectly activate the host’s immune–inflammatory system, which can damage tissues. They may stimulate the production of proinflammatory cytokines while decreasing the levels of anti-inflammatory biomolecules [13].

Inflammation plays an important role in the development and progression of HNCs [16]. It is often a response to the presence of cancer cells or toxins and carcinogens found in tobacco smoke, and may promote the growth and spread of cancer cells [16]. Inflammatory markers such as cytokines, chemokines, and growth factors are elevated in HNC patients, indicating the presence of chronic inflammation [17]. This chronic inflammation can also lead to tissue damage and impaired immune function, further contributing to the development of cancer [13].

The primary objective of this study was to meticulously assess the levels of an extensive panel of 48 inflammatory markers in patients diagnosed with HNC, with a particular focus on delineating the correlation between these biomarkers and smoking habits, a well-acknowledged risk factor in cancerogenesis. This comprehensive analysis included a diverse array of markers, such as cytokines, chemokines, growth factors, and other pivotal proteins involved in inflammation and tumor biology. This study represents an exploratory analysis, and as such, the results should be considered preliminary. They provide a foundation for future, more comprehensive research in this area.

## 2. Materials and Methods

### 2.1. Study Subjects

This study involved 50 patients diagnosed with primary HNC. The study participants were divided into two subgroups. The cigarette-smoking HNC group consisted of 25 patients. Eligibility for the HNC smoker group required participants to smoke cigarettes currently upon enrollment and to have maintained this habit for at least the past 10 years. The second subgroup comprised 25 patients classified in the non-smoking HNC group, each declaring they had never smoked cigarettes. To be included in the study, participants needed to have a diagnosis of malignant neoplasms of the lip, oral cavity, or pharynx (according to the International Classification of Diseases—11th Revision (ICD-11) codes: 2B60-2B69, 2B6A-2B6D), malignant neoplasms of the larynx (ICD-11 code: 2C23), or carcinoma in situ of the lip, oral cavity, or pharynx (ICD-11 code: 2E60.0) [18]. Patients with acute or chronic diseases, such as infectious, autoimmune, genetic, other cancers than HNC, and inflammatory conditions other than HNC, were excluded from the study. The decision to include or exclude a study participant was based on a medical interview with the patient and a review of the patient’s medical records. The patients in this study were treated at the Oncology Center of the Prof. Franciszek Łukaszczyk Memorial Hospital located in Bydgoszcz, Poland. The participants were enrolled in the study at the time of referral for planning radiotherapy with positron emission tomography–computed tomography (PET/CT). Inclusion in the study and the collection of test material took place before the start of the treatment. Patients were selected in such a way as to ensure the most uniform degree of disease progression among the subjects. The decision to include the patient in the study was made by a doctor employed at the Oncology Center of the Prof. Franciszek Łukaszczyk Memorial Hospital located in Bydgoszcz, Poland. A histopathological examination was performed on the patients, revealing that the study group consisted of patients with G1 squamous cell carcinoma, G2 squamous cell carcinoma, nonkeratinizing G2 squamous cell carcinoma, or G2 keratinizing squamous cell carcinoma.

The control group was composed of 30 healthy, non-smoking individuals with similar anthropometric characteristics to the patients from the HNC groups. Exclusion criteria for the control group included chronic or acute illnesses, such as cancer, diabetes, obesity, autoimmune disorders, and cardiometabolic disorders. Table 1 presents the results of the anthropometric analyses and clinical characteristics of the study participants.

All participants, from both the study and control groups, were given a questionnaire survey that included questions related to addictions and other factors that may predispose to the development of HNC. Participation in this research was voluntary, and it had no impact on the course of treatment. Consent was obtained from the participants prior to their inclusion in the study. The study was approved by the Bioethics Committee of the Nicolaus Copernicus University in Toruń, functioning at the Collegium Medicum in Bydgoszcz, Poland (consent no. KB 221/2018, approved on 23 March 2018).

### 2.2. Study Design

Qualified medical personnel from the Department of Nuclear Medicine at the Oncology Centre of Prof. Franciszek Łukaszczyk Memorial Hospital in Bydgoszcz, Poland collected blood samples from the median cubital vein between 7:00 a.m. and 9:00 a.m., after an overnight fast. A 6 mL polypropylene tube containing a clot activator and gel separator was used to collect each blood sample. The blood samples were transported under reduced temperature conditions to the laboratory of the Department of Medical Biology and Biochemistry, Faculty of Medicine, Ludwik Rydygier Collegium Medicum in Bydgoszcz of Nicolaus Copernicus University in Toruń, Poland, immediately after collection. Blood samples were subjected to centrifugation at 6000× *g* for 10 min at 4 °C to separate the blood serum from the blood clot. Following this step, the blood serum was aliquoted and placed in Eppendorf tubes. The serum samples were preserved at −80 °C for subsequent biochemical analysis.

### 2.3. Biochemical Analysis

A Bio-Plex Pro™ Human Cytokine Screening Panel, 48-plex (Bio-Rad Laboratories Inc., Hercules, CA, USA) kit was used to determine the serum concentrations of 48 analytes, namely cutaneous T-cell-attracting chemokine (CTACK); eosinophil chemotactic protein (Eotaxin); basic fibroblast growth factor (Basic FGF); granulocyte colony-stimulating factor (G-CSF); granulocyte-macrophage colony-stimulating factor (GM-CSF); growth-regulated alpha protein (GRO-α); hepatocyte growth factor (HGF); interferon alpha-2 (IFN-α2); interferon gamma (IFN-γ); interleukin 1 alpha (IL-1α); interleukin 1 beta (IL-1β); interleukin 1 receptor antagonist (IL-1ra); interleukin 2 (IL-2); interleukin 2 receptor alpha (IL-2Rα); interleukin 3 (IL-3); interleukin 4 (IL-4); interleukin 5 (IL-5); interleukin 6 (IL-6); interleukin 7 (IL-7); interleukin 8 (IL-8); interleukin 9 (IL-9); interleukin 10 (IL-10); interleukin 12 (p70) (IL-12 (p70)); interleukin 12 (p40) (IL-12 (p40)); interleukin 13 (IL-13); interleukin 15 (IL-15); interleukin 16 (IL-16); interleukin 17A (IL-17A); interleukin 18 (IL-18); interferon gamma-induced protein 10 (IP-10); leukemia inhibitory factor (LIF); monocyte chemoattractant protein-1 (MCP-1); monocyte chemoattractant protein-3 (MCP-3); macrophage colony-stimulating factor (M-CSF); macrophage migration inhibitory factor (MIF); monokine induced by gamma interferon (MIG); macrophage inflammatory protein-1 alpha (MIP-1α); macrophage inflammatory protein-1 beta (MIP-1β); beta-nerve growth factor (β-NGF); platelet-derived growth factor-BB (PDGF-BB); regulated on activation, normal T cell expressed and secreted (RANTES); stem cell factor (SCF); stem cell growth factor beta (SCGF-β); stromal cell-derived factor 1 alpha and beta (SDF-1α+β); tumor necrosis factor alpha (TNF-α); tumor necrosis factor beta (TNF-β); TNF-related apoptosis-inducing ligand (TRAIL); and vascular endothelial growth factor (VEGF). These analytes were determined with the use of a commercially available research kit. The study followed the manufacturer’s instructions for all analyses. The enzyme immune assay kit used in the study included standard concentration analytes, blank and control samples, and other necessary reagents for the analysis. Bio-Plex Multiplex immunoassay is a technique used for the detection and quantification of multiple protein biomarkers simultaneously in a single sample. It involves the use of magnetic beads that are coated with specific antibodies to capture and detect different proteins in a complex mixture. The volume of serum used for the analysis is 15 µL, and at least 50 magnetic beads in each region are required to complete the sample analysis. Subsequently, the proteins captured by the beads bind to detection antibodies. The formation of this “sandwich” complex is then targeted by a streptavidin-phycoerythrin (SA-PE) conjugate, introducing fluorescence through phycoerythrin as a fluorescent indicator or reporter for the detection phase. The next step is the addition of reporter dye to the reaction mixture and a reading using a laser-based system that detects the amount of fluorescence emitted. Fluorescence was measured using the Bio-Plex^®^ 200 system (Bio-Rad Laboratories Inc., Hercules, CA, USA). This system employs dual-laser technology for precise measurement. A red laser (635 nm) identifies each bead, determining the analyte based on the bead’s fluorescence, while a green laser (532 nm) quantifies the reporter signal from the phycoerythrin, correlating directly with the analyte’s concentration. This process is managed by a high-speed digital processor, and results are presented with the use of Bio-Plex Manager™ 6.2 Software (Bio-Rad Laboratories Inc., Hercules, CA, USA) as median fluorescence intensity (MFI), allowing for an accurate concentration determination based on the MFI. To ensure accuracy and reliability, each sample concentration underwent duplicate measurements. Subsequently, the average of the two measurements was calculated and included in our analysis. This meticulous approach aimed to minimize variability and uphold the highest standard of data integrity. The results were reported in picograms per milliliter (pg/mL) or nanograms per milliliter (ng/mL).

### 2.4. Statistical Analysis

In this research, data analysis was carried out using Statistica 13.3 by TIBCO Software Inc. (Palo Alto, CA, USA) and Python 3.8.10 from the Python Software Foundation (Wilmington, DE, USA), incorporating libraries such as pandas (1.4.3), matplotlib (3.1.3), and scipy (1.10.1). The data were presented as mean values, standard error of the mean (SEM), medians, and interquartile ranges (IQR). Group equivalences were evaluated using the chi-square test. The Shapiro–Wilk test assessed data normality, while Levene’s test checked for the homogeneity of variances. For comparisons between two independent groups, the Student’s *t*-test was applied, and the chi-square test of independence was used for categorical data. Primary analyses involved a one-way ANOVA with a Tukey HSD post hoc test for the data meeting both the normal distribution and homogeneity criteria. In cases of normal distribution without homogeneity, a T2 Tamhane post hoc test followed a one-way ANOVA. For non-normally distributed data, analysis began with the Kruskal–Wallis test, succeeded by the Mann–Whitney U test with Bonferroni adjustment for multiple comparisons. Spearman’s correlation coefficient was utilized to explore correlations between variables. This comprehensive statistical framework was crucial for the precise interpretation of the results, with statistical significance determined at a *p*-value less than 0.05.

## 3. Results

There were no significant differences between the groups for gender, age, body mass, height, and BMI. Regrettably, the datasets related to physical activity and alcohol intake do not feature groups of adequate size to facilitate the execution of chi-square statistical analyses. This limitation arises due to the insufficient number of observations within each category, which is essential for the reliability and validity of chi-square test results. As a direct consequence of this constraint, we have decided to exclude the *p*-values associated with these specific parameters from Table 1. This decision was made to ensure the integrity and accuracy of our statistical reporting, acknowledging that including *p*-values for underpowered tests could lead to misleading interpretations.

The laboratory results are detailed in Table 2. The comprehensive statistical analysis conducted as part of our study revealed significant disparities in the levels of a broad spectrum of inflammatory markers, including CTACK, Basic FGF, G-CSF, GM-CSF, HGF, IFN-α2, IFN-γ, IL-1α, IL-1β, IL-1ra, IL-2, IL-2Rα, IL-3, IL-6, IL-7, IL-8, IL-9, IL-10, IL-12 (p70), IL-12 (p40), IL-13, IL-15, IL-17A, IL-18, IP-10, LIF, MCP-1, MCP-3, M-CSF, MIF, MIG, MIP-1α, MIP-1β, PDGF-BB, RANTES, SCF, SCGF-β, SDF-1α+β, TNF-α, TNF-β, TRAIL, and VEGF, between the distinct groups involved in the study, including HNC patients with varying smoking histories and healthy controls.

In the event that the analytical methods used did not allow for the determination of the parameter in the test material due to its low circulating level, the lower limit of quantification (LLOQ) value was adopted as the test result. The LLOQ value was read from the documentation attached to the ready-to-use Bio-Plex Pro™ Human Cytokine Screening Panel, 48-plex kit (Bio-Rad Laboratories Inc., Hercules, CA, USA). The LLOQ value was used for analytes GM-CSF, IFN-α2, IL-2, IL-3, IL-6, IL-7, IL-10, IL-12 (p70), IL-12 (p40), IL-15, IL-17A, MCP-3, and VEGF in a group of healthy subjects.

Building upon the initial observations, Table 3 meticulously details the outcomes of the subsequent post hoc analysis, specifically designed to thoroughly investigate and clarify the significant associations and disparities unveiled in the preliminary results. A comparison between the smoking and non-smoking HNC patients revealed a significantly lower concentration of M-CSF in smokers with cancer. The analysis showed that smokers with HNC exhibited significantly elevated levels of CTACK, Eotaxin, Basic FGF, G-CSF, GM-CSF, GRO-α, HGF, IFN-α2, IFN-γ, IL-1α, IL-1β, IL-1ra, IL-2, IL-2Rα, IL-3, IL-4, IL-5, IL-6, IL-7, IL-8, IL-9, IL-12 (p70), IL-12 (p40), IL-13, IL-16, IL-17A, IL-18, IP-10, LIF, MCP-1, MCP-3, M-CSF, MIF, MIG, MIP-1α, MIP-1β, β-NGF, PDGF-BB, RANTES, SCF, SCGF-β, SDF-1α+β, TNF-α, TNF-β, TRAIL, and VEGF compared to the controls. In the comparison between the non-smoking HNC and control groups, significant differences were observed in the levels of various inflammatory markers, including CTACK, Eotaxin, Basic FGF, G-CSF, GM-CSF, GRO-α, HGF, IFN-α2, IFN-γ, IL-1α, IL-1β, IL-1ra, IL-2, IL-2Rα, IL-3, IL-4, IL-5, IL-6, IL-7, IL-8, IL-9, IL-10, IL-12 (p70), IL-12 (p40), IL-13, IL-15, IL-16, IL-17A, IL-18, IP-10, LIF, MCP-1, MCP-3, M-CSF, MIF, MIG, MIP-1α, MIP-1β, β-NGF, RANTES, SCF, SCGF-β, SDF-1α+β, TNF-α, TNF-β, TRAIL, and VEGF. 

**Figure 1:**
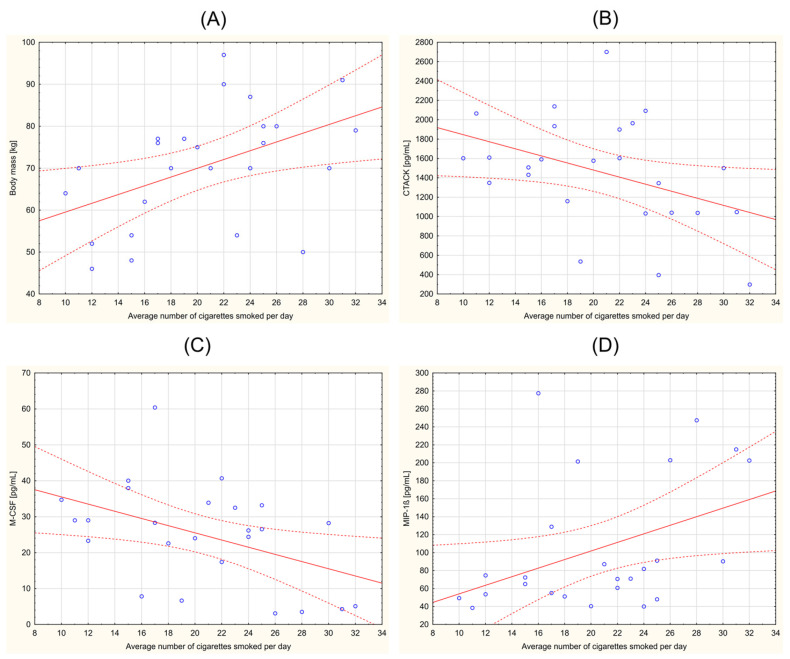
Correlations in the head and neck cancer (HNC) smoking group. Average number of cigarettes smoked per day vs. (**A**) body mass (r = 0. 469; *p* = 0. 018); (**B**) CTACK (r = −0. 407; *p* = 0.044); (**C**) M-CSF (r = −0.450; *p* = 0.024); and (**D**) MIP-1β (r = 0.413; *p* = 0.040). Abbreviations used: CTACK: cutaneous T-cell-attracting chemokine; M-CSF: macrophage colony-stimulating factor; MIP-1β: macrophage inflammatory protein 1 beta. *p* < 0.05 was considered statistically significant.

The statistical analysis in this study comprehensively evaluated the correlations between various clinical parameters across the participant groups. Notably, within the HNC smoking group, significant correlations emerged. A significant positive correlation was observed between the average number of cigarettes smoked per day and body mass (r = 0.469; *p* = 0.018). Additionally, significant negative correlations were identified between the average number of cigarettes smoked and both CTACK (r = −0.407; *p* = 0.044) and M-CSF (r = −0.450; *p* = 0.024). In contrast, a positive correlation was found between smoking and MIP-1β (r = 0.413; *p* = 0.040). Figure 1 provides Pearson’s correlation diagrams for these parameters within the HNC smoking group, offering a visual representation of the observed statistical relationships. Additional results of the Spearman’s rank correlation analysis of the studied parameters in both the smoking and non-smoking HNC groups are presented in the Supplementary Materials (Figure S1: Spearman’s rank correlation analysis).

## 4. Discussion

The relationship between inflammation and neoplastic diseases, including HNCs, has been the subject of much research in recent years [19,20]. Our study revealed that out of the 48 analyzed biomarkers, 41 exhibited statistically significant differences in their levels when comparing non-smoking HNC patients to the control group. This suggests a significant disturbance in the inflammatory state during HNCs. However, little is known about the relationship between the profile of inflammatory markers and HNCs in both smoking and non-smoking patients. Such a comparison would allow for a better understanding of the negative impact of cigarette smoking on the carcinogenesis of HNCs and, in a broader perspective, may improve disease detection and prognosis.

Inflammation plays a crucial role in the local environment of tumors, with mediators and cellular effectors acting as important constituents [21]. Persistent inflammation in the tumor microenvironment has several tumor-promoting effects, such as aiding in malignant cell proliferation and survival, promoting angiogenesis and metastasis, subverting adaptive immune responses, and altering responses to hormones and chemotherapy [22,23]. A diverse range of inflammatory cells, including T lymphocytes (occasionally B cells), dendritic cells, macrophages, monocytes, neutrophils, and natural killer (NK) cells, are present within the tumor microenvironment [24]. Smoking is also associated with an increase in the level of proinflammatory markers as well as a decrease in the concentration of molecules that reduce inflammation [25]. The up-regulation of various proinflammatory genes appears to be the main cellular mechanism behind the proinflammatory effects of cigarette smoke and its constituents, which is activated by the nuclear factor kappa-light-chain-enhancer of activated B cells (NF-κB) pathway [26]. Smoking not only causes inflammation but also leads to an overproduction of reactive oxygen species (ROS) and consequently augmented oxidative stress [27]. The large number of harmful chemicals in tobacco smoke can trigger an immune response and damage cells and tissues [15].

Smoking significantly elevates the risk of HNC and detrimentally influences the success of treatment protocols [28,29,30,31]. The connection between HNC and cigarette smoking is a critical aspect of understanding and managing this type of cancer [32]. According to the meta-analysis by Koyanagi et al. [29] in the Japanese population, cigarette smoking is strongly associated with an increased risk of developing HNC, highlighting the significant impact of tobacco use on cancer incidence in this demographic. Merlano et al. [33] indicated that smokers with head and neck squamous cell carcinoma generally have worse outcomes compared to non-smokers and those who have quit smoking. 

HNCs are characterized by their inflammatory and aggressive behavior, with the expression of various cytokines and growth factors that drive inflammation [34]. These molecules are key in fostering tumor growth by facilitating tissue remodeling and angiogenesis, and by supporting the survival of tumor cells and their resistance to chemotherapy [35]. They achieve this through both autocrine and paracrine actions. Furthermore, these cytokines and growth factors trigger critical signaling pathways like NF-κB, Janus kinase/signal transducer and activator of transcription (JAK-STAT), and phosphatidylinositol-3-kinase and the mammalian target of rapamycin (PI3K/Akt/mTOR), which are instrumental in regulating genes that control tumor growth, survival, and the sensitivity to chemotherapy [35]. In the study by Saroul et al. [36], the authors indicated that the prognosis for HNC depends on various factors, with malnutrition and inflammation being significant yet potentially modifiable aspects. Allen et al. [37] investigated the potential of cytokines and growth factors as early biomarkers for treatment response and survival in patients with advanced head and neck squamous cell carcinomas. It focused on the longitudinal changes in serum levels of specific factors like IL-6, IL-8, VEGF, HGF, and GRO-α in 30 patients undergoing chemoradiation therapy for stage III/IV oropharyngeal squamous cell carcinomas. This prospective trial revealed that these biomarkers are closely linked to how patients respond to therapy and their overall survival prospects, suggesting their potential utility in predicting treatment outcomes and guiding clinical decisions for individuals with cancer. 

In our study, we observed that, among 48 analytes, one parameter, namely M-CSF, significantly differed in levels between smokers and non-smokers with HNC. Considering the variability in disease progression among patients, further analysis is warranted to determine whether the observed difference in M-CSF levels between smokers and non-smokers with HNC may be attributed to variations in disease status. M-CSF is essential for controlling the differentiation, survival, proliferation, and renewal of both monocytes and macrophages [38]. M-CSF is a glycoprotein that is synthesized and secreted by various cell types, including osteoblasts, fibroblasts, endothelial cells, and macrophages [39]. It exists in several isoforms, including a membrane-bound form and a soluble form that is released into the extracellular environment [40]. M-CSF has been found to have various properties, including immunomodulatory and angiogenic qualities [41]. It has been implicated in a variety of physiological and pathological processes, including inflammation, tissue repair, and cancer [38]. M-CSF signaling pathways can activate multiple downstream signaling cascades that regulate various cellular processes. The binding of M-CSF to its receptor, the M-CSF receptor (M-CSFR or CSF1R), leads to the autophosphorylation of the receptor’s intracellular tyrosine residues and the recruitment and activation of several downstream signaling molecules [42]. One of the major signaling pathways activated by M-CSFR is the PI3K/Akt/mTOR pathway [43]. This pathway plays a key role in regulating cell survival, proliferation, and metabolism. The mitogen-activated protein kinases/extracellular signal-regulated kinases (MAPK/ERK) and JAK/STAT pathways are other major signaling pathways activated by M-CSF [44,45]. In HNC, colorectal, pancreatic, and prostate tumors, M-CSF expression is upregulated, leading to the recruitment and activation of tumor-associated macrophages (TAMs) [41,46]. TAMs can promote tumor growth, invasion, and metastasis by secreting factors that stimulate angiogenesis, suppress the immune response, and remodel the extracellular matrix [47]. Additionally, M-CSF has been shown to promote cancer cell survival and invasion in some types of cancer, making it a potential target for cancer therapy [41]. In our study, the concentration of M-CSF was lower in the smoking HNC group than in non-smoking patients. A significantly higher level of M-CSF was also observed in the non-smoking HNC group compared to the control group. McDermott et al. [46] in their study observed that the levels of M-CSF were significantly higher in patients with newly diagnosed tumors of the head and neck, men with prostate cancer metastatic to bone, and women with advanced metastatic breast cancer when compared to those with newly diagnosed breast tumors. The effect of cigarette smoking on M-CSF levels in HNCs remains unknown. In a study by Köttstorfer et al. [48], the mean overall serum concentration of M-CSF was significantly higher in non-smoking individuals, women, and older patients. The study group consisted of 51 patients with long bone fractures. We believe the role of M-CSF in the progression of HNC among smokers and non-smokers remains uncertain. It is unclear whether M-CSF contributes to disease development or is altered by the disease’s progression. Further studies with larger patient cohorts are required to better comprehend the correlation between the expression of M-CSF and smoking. 

Elevated IL-10 levels were observed in both HNC patient groups compared to controls, with a statistically significant increase noted exclusively in the non-smoking HNC group. Between the HNC groups themselves, no significant variance in IL-10 levels was detected. IL-10 plays a nuanced role in cancer, functioning as a critical regulator between homeostatic immunity and inflammation. IL-10 is mainly an anti-inflammatory cytokine that inhibits the production of selected proinflammatory cytokines [49]. As a cytokine, IL-10 mediates intercellular signaling that can influence immune and inflammatory responses, making its modulation a promising avenue for cancer immunotherapy. The complexity of IL-10’s function is highlighted by its paradoxical roles in cancer, where it can exhibit both pro- and antitumor effects within the tumor microenvironment [50,51]. This dual nature adds a layer of complexity to its prognostic value and therapeutic potential in cancer treatment. The interaction of IL-10 with the immune system is particularly significant, as it has been shown to have immune-stimulatory functions that are crucial for T-helper cell activities and T-cell immune surveillance [52]. This relationship underscores the potential of IL-10 to enhance tumor-specific immune surveillance while also controlling pathogenic inflammation, which could contribute to its emerging role as a key player in cancer pathology. IL-10 has been the subject of limited studies in patients with HNC. According to the study by Bornstein et al. [53], molecular analyses of HNC tumors that progressed despite the treatment have identified the IL-10 and integrin signaling pathways as significantly associated with cancer progression. The meta-analysis by Huang et al. [54] aimed to clarify the relationship between the IL-10 rs1800896 polymorphism, HNC risk, and its clinical stages. Analyzing six case–control studies with a total of 1781 patients and 1978 controls, the study found a significant association between the rs1800896 polymorphism and an increased risk of HNC. The conclusion was that the interleukin-10 rs1800896 polymorphism is significantly linked to the risk of developing HNC but does not correlate with the disease’s clinical stages. The findings from our research suggest that smoking may detrimentally affect the immune system’s functionality. This could explain why both of the HNC groups exhibited higher IL-10 levels than the control group, though only the difference in non-smokers with HNC reached statistical significance. This implies that the immunomodulatory effects of IL-10, crucial for controlling inflammation and promoting immune tolerance, might be compromised in smokers. Consequently, this could impact the body’s ability to mount an effective immune response against HNC, underscoring the complex relationship between smoking, immune regulation, and cancer progression. Further investigation is needed to understand how smoking affects other immune parameters in HNC patients, which could offer deeper insights into how smoking influences disease development and progression.

In our study, we observed significantly higher levels of IL-15 in non-smoking HNC patients compared to the control group. Although IL-15 concentrations appeared elevated in smokers compared to the control group, the statistical analysis did not confirm this difference as significant. Essentially, the levels of this analyte were comparable between the smoking and non-smoking HNC groups. IL-15 plays a pivotal role in the immune response against cancer [55]. As a cytokine in the common gamma-chain family, IL-15 is essential for the development, proliferation, and activity of key immune cells such as T cells, B cells, and NK cells [55,56]. Its ability to enhance the immune system’s antitumor activity positions it as a focal point in the realm of cancer immunotherapy. The significance of IL-15 is particularly noted in its promotion of NK cell activation and proliferation [57]. NK cells are instrumental in the innate immune defense against tumors, and their stimulation by IL-15 facilitates the direct targeting and elimination of cancer cells [57]. Furthermore, IL-15 is crucial for the sustenance and functionality of memory CD8+ T cells, which are vital for long-term immune surveillance and the prevention of tumor relapse [58]. The therapeutic potential of IL-15 in cancer treatment is highlighted by its capacity to alter the tumor microenvironment from one that supports tumor growth to one that is inhospitable to cancer cells. By amplifying the cytotoxic action of immune cells against tumors, IL-15 emerges as a promising candidate for cancer therapy, particularly in cancers known for immune evasion, such as HNC [59]. The scientific literature currently lacks data on how IL-15 levels change over time in patients with HNC, particularly when comparing smokers to non-smokers. The absence of a statistically significant difference in IL-15 levels between the smoking HNC group and the control group, as observed in our study, may serve as an unfavorable prognostic indicator given the critical role of IL-15 in combating tumor growth. In contrast, the elevated levels of IL-15 detected in the non-smoking HNC group could potentially confer a more robust immune defense against cancer, facilitating a more effective battle against the disease. This disparity underscores the potential impact of smoking on the immune system’s efficacy in cancer surveillance and response, highlighting the need for further research to elucidate the mechanisms through which smoking may influence IL-15 levels and, by extension, the immune response to HNC.

Our analysis found that PDGF-BB levels in smokers with HNC were significantly elevated when compared to those in the control group. While it appeared that PDGF-BB levels in non-smokers with HNC were also higher than in controls, this increase did not reach statistical significance. PDGF-BB concentrations in HNC patients do not significantly differ based on smoking status. PDGF-BB is integral in various biological processes, including cell growth, blood vessel formation, and wound healing [60,61]. PDGF-BB plays a crucial role in cancer development by influencing proliferation, metastasis, invasion, and angiogenesis [62,63,64]. It functions through various signaling pathways, including the PI3K/Akt and MAPK/ERK pathways [65]. Targeting the PDGF/PDGF receptor pathway has shown effectiveness in cancer therapy, leading to significant advancements in this field [63]. In the study by Er et al. [66], the impact of PDGF-BB on the radiotherapy response in esophageal squamous cell carcinoma was examined. Despite radiotherapy being a primary treatment for advanced esophageal squamous cell carcinoma, the search for reliable biological markers for prognosis remains ongoing. It has been found that PDGF-BB is linked to poor outcomes across various cancers. The analysis of serum from 68 patients undergoing radiotherapy showed that reduced PDGF-BB levels could indicate better survival rates. Targeting *PDGFB* reduced cancer cell growth and increased radiation sensitivity, suggesting the potential of PDGF-BB as a predictive marker for esophageal squamous cell carcinoma radiotherapy response. In our study, we noted significantly elevated PDGF-BB levels in the smoking HNC group compared to healthy individuals. However, between non-smokers and controls, the differences were not statistically significant, suggesting that cigarette smoking might elevate PDGF-BB levels, potentially indicating a poorer prognosis.

Although our study is constrained by a relatively small sample size, it is essential to recognize the novelty of our research approach. No previous investigations have concurrently assessed such a comprehensive spectrum of inflammatory markers, including cytokines, chemokines, and growth factors, particularly in a European HNC patient cohort. The limited sample size stems from the stringent inclusion and exclusion criteria designed to ensure the homogeneity of the study groups, which, while advantageous for controlling confounding variables, inevitably restricts the participant pool. This rigorous selection process, beneficial for the integrity of the study, poses challenges in accurately representing the broader population, potentially introducing biases, and affecting the statistical power needed to detect significant differences between groups. Despite these challenges, it is commendable that advanced statistical analyses were employed to discern statistically significant differences across the study’s variables, demonstrating the robustness of our findings within the constraints imposed by the sample size. However, the results warrant cautious interpretation and underscore the necessity for subsequent research endeavors involving larger cohorts to corroborate and build upon these preliminary insights. This study’s contributions are significant, shedding light on the intricate role of inflammation in HNC and emphasizing the impact of smoking as a pivotal risk factor for cancer development. By offering a detailed exploration of inflammatory profiles in HNC patients, our research paves the way for future studies to delve deeper into targeted therapies and preventive strategies, ultimately aiming to enhance patient outcomes in this domain. 

**Figure 2:**
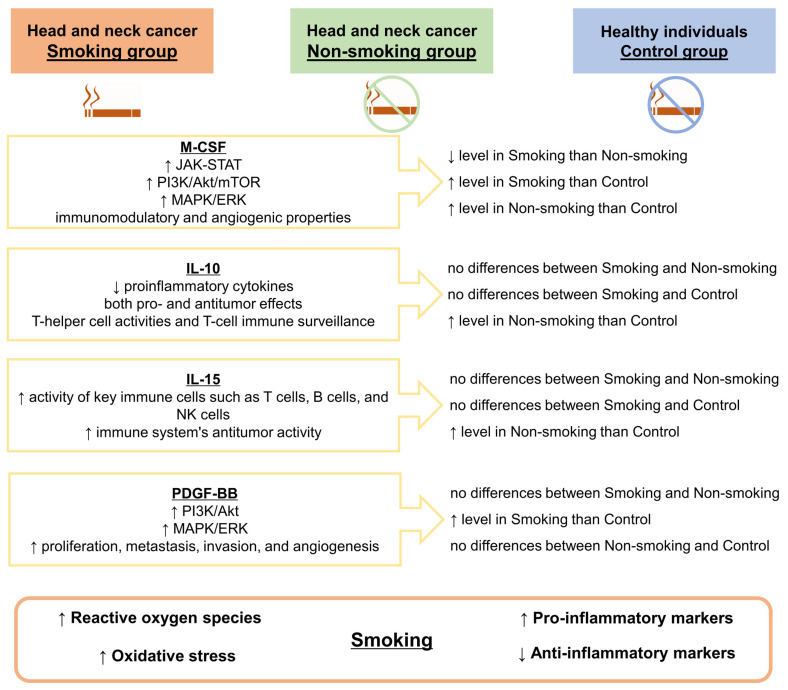
Effects of cigarette smoking on the levels of M-CSF, IL-10, IL-15, and PDGF-BB in the course of head and neck cancer. The main sources of cytokines and the signaling pathways activated by them are included. Abbreviations used: IL-10: interleukin 10; IL-15: interleukin 15; JAK-STAT: Janus kinase-signal transducer and activator of transcription pathway; M-CSF: macrophage colony-stimulating factor; MAPK/ERK: mitogen-activated protein kinase/extracellular signal-regulated kinase pathway; NK cells: natural killer cells; PDGF-BB: platelet-derived growth factor BB; PI3K/Akt/mTOR: phosphoinositide 3-kinase/Protein kinase B/mammalian target of rapamycin. Symbols used: ↑: higher; ↓: lower.

Figure 2 summarizes the results of our study, presenting potential links between M-CSF, IL-10, IL-15, and PDGF-BB, and in the course of HNCs, taking into account the impact of tobacco smoking by patients.

## 5. Conclusions

The findings of our study indicate that smoking not only amplifies the inflammatory response but also contributes to creating a milieu conducive to cancer development and progression in HNC. Smoking is associated with elevated levels of M-CSF and the activation of pathways that promote immunomodulation and angiogenesis, indicating a more aggressive cancer phenotype. In contrast, non-smoking HNC patients display higher levels of immune-enhancing cytokines like IL-10 and IL-15, which might suggest a more effective antitumor response. PDGF-BB levels, implicated in cancer proliferation and metastasis, are also higher in smokers, further linking smoking with aggressive cancer progression. These findings highlight the complex interplay between smoking, inflammatory response, and cancer progression in HNC, warranting further investigation for targeted therapy. Our exploratory analysis contributes novel insights into the nuanced relationship between tobacco smoking, inflammatory biomarkers, and HNC progression, emphasizing the differential impact of smoking on the inflammatory response associated with cancer development. By elucidating the specific inflammatory markers influenced by smoking, such as elevated M-CSF in smokers and higher IL-10 and IL-15 levels in non-smoking HNC patients, this research provides a deeper understanding of the role of smoking in modulating the inflammatory landscape of HNC. These findings not only enhance our comprehension of the pathophysiological mechanisms linking smoking with HNC but also underscore the potential for targeted therapeutic interventions that modulate the inflammatory response in HNC patients. Further research is imperative to elucidate the intricate relationships between smoking, inflammatory biomarkers, and HNC progression.

## Figures and Tables

**Table 1:** The anthropometric and clinical characteristics of both the head and neck cancer (HNC) patients and healthy volunteers (control group).

Parameter		HNC		Control	*p*-Value	Power of a Test
		Smoking	Non-Smoking			
n (Female/Male)		25 (11/14)	25 (10/15)	30 (13/17)	-	-
Age [years]	Mean	61.600	61.920	62.467	0.8247	0.1497
	SEM	1.377	1.008	0.655		
	Median	60.000	62.000	63.000		
	IQR	6.000	5.000	5.000		
Body Mass [kg]	Mean	70.600	70.924	67.500	0.2325	1.0000
	SEM	2.805	2.288	1.251		
	Median	70.000	73.000	67.000		
	IQR	17.000	18.000	11.000		
Height [m]	Mean	1.69	1.71	1.69	0.5397	0.3995
	SEM	0.0181	0.0124	0.0101		
	Median	1.69	1.73	1.70		
	IQR	0.12	0.08	0.10		
BMI [kg/m^2^]	Mean	24.369	24.037	23.369	0.0931	0.8827
	SEM	0.584	0.606	0.279		
	Median	25.059	24.676	23.939		
	IQR	4.444	5.897	2.227		
Average number of cigarettes smoked per day [n]	Mean	20.600	0	0	-	-
	SEM	1.261	0	0		
	Median	21.000	0	0		
	IQR	9.000	0	0		
	Min	10	0	0		
	Max	32	0	0		
Frequency of strong alcohol consumption [n]	A few times a week	1	1	0	-	-
	Once a week	10	7	4		
	Once every two weeks	6	7	5		
	Less often	5	6	10		
	No alcohol consumption	3	4	11		
Frequency of physical activity [n]	Every day	0	1	3	-	-
	A few times a week	1	3	9		
	Once a week	3	4	10		
	Less often	4	4	6		
	No physical activity	17	13	2		

Abbreviations used: BMI: body mass index; IQR: interquartile range; SEM: standard error of mean. *p* < 0.05 was considered statistically significant.

**Table 2:** Results of biochemical analyses in the smoking head and neck cancer (HNC), non-smoking HNC, and the healthy control groups. *p* < 0.05 was considered statistically significant.

Parameter		HNC		Control n = 30	*p*-Value	Power of a Test
		Smoking n = 25	Non-Smoking n = 25			
CTACK [pg/mL]	Mean	1458.197	1752.868	902.949	<0.0001	1.0000
	SEM	113.280	149.048	57.056		
	Median	1507.790	1687.180	880.405		
	IQR	853.170	856.340	414.760		
Eotaxin [pg/mL]	Mean	139.020	127.476	102.908	0.2146	1.0000
	SEM	17.180	12.629	10.215		
	Median	125.250	114.190	90.440		
	IQR	126.000	46.100	54.570		
Basic FGF [pg/mL]	Mean	58.025	67.913	28.800	<0.0001	1.0000
	SEM	4.713	3.878	2.886		
	Median	66.490	66.490	26.730		
	IQR	23.460	13.550	9.320		
G-CSF [pg/mL]	Mean	120.454	150.841	36.364	<0.0001	1.0000
	SEM	12.121	13.436	6.013		
	Median	109.420	157.250	32.885		
	IQR	97.370	64.310	33.610		
GM-CSF [pg/mL]	Mean	1.244	1.151	0.480	0.0344	0.7426
	SEM	0.343	0.287	0.000		
	Median	0.480	0.480	0.480		
	IQR	0.000	0.000	0.000		
GRO-α [pg/mL]	Mean	785.867	682.210	649.428	0.4965	1.0000
	SEM	54.894	45.123	62.209		
	Median	720.350	705.670	662.705		
	IQR	295.730	216.280	529.260		
HGF [pg/mL]	Mean	865.029	1264.327	288.109	<0.0001	1.0000
	SEM	124.092	166.989	17.531		
	Median	765.640	1081.750	276.405		
	IQR	660.440	1006.650	82.460		
IFN-α2 [pg/mL]	Mean	20.631	19.700	0.950	<0.0001	1.0000
	SEM	0.944	1.377	0.000		
	Median	19.960	19.960	0.950		
	IQR	4.370	7.900	0.000		
IFN-γ [pg/mL]	Mean	16.040	20.529	7.042	<0.0001	1.0000
	SEM	2.705	2.609	0.463		
	Median	11.720	15.650	6.595		
	IQR	12.960	15.120	2.510		
IL-1α [pg/mL]	Mean	17.118	17.347	4.671	<0.0001	1.0000
	SEM	4.142	3.371	0.654		
	Median	11.260	13.250	3.730		
	IQR	17.130	23.930	0.000		
IL-1β	Mean	3.396	3.704	0.498	<0.0001	1.0000
	SEM	0.499	0.482	0.078		
	Median	3.050	3.090	0.290		
	IQR	2.020	2.200	0.000		
IL-1ra [pg/mL]	Mean	505.377	561.774	169.045	<0.0001	1.0000
	SEM	71.457	80.716	13.833		
	Median	398.820	528.540	156.015		
	IQR	267.100	506.610	67.130		
IL-2 [pg/mL]	Mean	3.129	4.931	1.290	0.0002	1.0000
	SEM	0.687	1.190	0.000		
	Median	1.290	1.290	1.290		
	IQR	2.010	5.030	0.000		
IL-2Rα [pg/mL]	Mean	84.220	110.756	45.417	<0.0001	1.0000
	SEM	6.701	9.901	3.019		
	Median	86.120	102.280	41.550		
	IQR	46.190	59.580	17.010		
IL-3 [pg/mL]	Mean	1.362	1.699	0.130	<0.0001	0.9976
	SEM	0.335	0.187	0.000		
	Median	0.620	1.690	0.130		
	IQR	1.860	0.980	0.000		
IL-4 [pg/mL]	Mean	2.248	2.383	1.988	0.5138	0.2324
	SEM	0.100	0.231	0.192		
	Median	2.330	2.250	2.110		
	IQR	0.600	1.280	1.360		
IL-5 [pg/mL]	Mean	3.647	3.647	3.647	0.9766	0.0500
	SEM	0.014	0.014	0.012		
	Median	3.630	3.630	3.630		
	IQR	0.000	0.000	0.000		
IL-6 [pg/mL]	Mean	4.487	7.092	0.380	<0.0001	1.0000
	SEM	1.037	1.293	0.000		
	Median	2.410	5.540	0.380		
	IQR	6.040	12.830	0.000		
IL-7 [pg/mL]	Mean	16.690	15.493	1.920	<0.0001	1.0000
	SEM	2.198	1.763	0.000		
	Median	17.090	17.090	1.920		
	IQR	10.590	9.980	0.000		
IL-8 [pg/mL]	Mean	16.126	16.941	7.337	0.0008	1.0000
	SEM	3.140	2.701	0.635		
	Median	11.020	14.290	6.415		
	IQR	7.380	18.750	2.900		
IL-9 [pg/mL]	Mean	123.837	88.550	502.017	<0.0001	1.0000
	SEM	15.556	9.262	3.948		
	Median	90.110	81.090	506.195		
	IQR	33.570	36.240	36.050		
IL-10 [pg/mL]	Mean	1.456	1.796	1.060	0.0053	0.6258
	SEM	0.280	0.404	0.000		
	Median	1.060	1.060	1.060		
	IQR	0.000	0.010	0.000		
IL-12 (p70) [pg/mL]	Mean	3.824	3.341	1.430	0.0004	1.0000
	SEM	0.869	1.048	0.000		
	Median	1.430	1.430	1.430		
	IQR	3.120	0.870	0.000		
IL-12 (p40) [pg/mL]	Mean	167.134	205.251	14.680	<0.0001	1.0000
	SEM	25.555	28.714	0.000		
	Median	161.010	178.960	14.680		
	IQR	110.890	147.680	0.000		
IL-13 [pg/mL]	Mean	3.340	4.489	0.928	<0.0001	1.0000
	SEM	0.683	1.000	0.306		
	Median	2.220	2.880	0.310		
	IQR	5.020	4.240	0.000		
IL-15 [pg/mL]	Mean	52.302	60.704	12.420	0.0458	1.0000
	SEM	19.055	21.310	0.000		
	Median	12.420	12.420	12.420		
	IQR	0.000	0.000	0.000		
IL-16 [pg/mL]	Mean	67.369	82.065	48.114	0.6491	1.0000
	SEM	13.513	17.297	3.614		
	Median	51.610	54.770	44.055		
	IQR	108.310	121.390	21.650		
IL-17A [pg/mL]	Mean	10.338	12.556	2.440	<0.0001	1.0000
	SEM	1.171	1.775	0.000		
	Median	11.580	12.420	2.440		
	IQR	12.260	14.360	0.000		
IL-18 [pg/mL]	Mean	72.940	97.220	35.260	<0.0001	1.0000
	SEM	9.330	10.921	3.650		
	Median	57.420	84.630	31.030		
	IQR	40.020	30.850	26.200		
IP-10 [pg/mL]	Mean	1250.649	1459.909	495.298	<0.0001	1.0000
	SEM	215.017	159.842	69.284		
	Median	985.360	1400.980	406.375		
	IQR	1323.870	734.530	161.390		
LIF [pg/mL]	Mean	45.032	54.508	15.098	0.0004	1.0000
	SEM	7.005	9.041	2.691		
	Median	45.330	57.260	10.345		
	IQR	61.310	73.010	18.400		
MCP-1 [pg/mL]	Mean	84.806	83.477	37.471	<0.0001	1.0000
	SEM	10.490	8.800	3.939		
	Median	83.460	78.570	31.470		
	IQR	87.060	34.420	27.130		
MCP-3 [pg/mL]	Mean	2.112	2.598	0.480	<0.0001	0.9999
	SEM	0.441	0.449	0.000		
	Median	0.620	2.640	0.480		
	IQR	3.710	3.570	0.000		
M-CSF [pg/mL]	Mean	24.916	37.500	5.783	<0.0001	1.0000
	SEM	2.802	3.498	0.632		
	Median	26.540	35.250	5.295		
	IQR	15.820	19.300	3.360		
MIF [pg/mL]	Mean	1771.194	2417.451	591.863	<0.0001	1.0000
	SEM	379.186	426.807	102.379		
	Median	1058.540	1718.620	456.030		
	IQR	1394.900	2148.950	422.190		
MIG [pg/mL]	Mean	771.724	1041.315	145.324	<0.0001	1.0000
	SEM	129.188	145.282	25.763		
	Median	705.950	853.010	103.865		
	IQR	763.680	864.400	70.850		
MIP-1α [pg/mL]	Mean	3.761	4.362	1.758	<0.0001	1.0000
	SEM	0.395	0.354	0.199		
	Median	3.470	4.360	1.490		
	IQR	2.110	1.890	0.620		
MIP-1β [pg/mL]	Mean	104.682	68.332	411.826	<0.0001	1.0000
	SEM	14.570	5.921	4.481		
	Median	72.370	61.810	416.440		
	IQR	75.160	29.150	31.030		
β-NGF [pg/mL]	Mean	2.164	1.480	2.137	0.6782	0.6390
	SEM	0.780	0.625	0.762		
	Median	0.470	0.470	0.470		
	IQR	1.720	0.670	0.000		
PDGF-BB [pg/mL]	Mean	6882.342	6390.921	3455.909	0.0225	1.0000
	SEM	982.017	996.719	279.008		
	Median	5566.670	4419.040	3288.955		
	IQR	4841.070	5696.230	2451.180		
RANTES [ng/mL]	Mean	33.573	30.619	12.535	<0.0001	1.0000
	SEM	4.136	4.304	0.994		
	Median	28.127	24.290	11.121		
	IQR	28.106	26.923	3.176		
SCF [pg/mL]	Mean	79.349	95.429	62.904	0.0009	1.0000
	SEM	5.175	8.688	3.130		
	Median	81.580	88.430	62.630		
	IQR	40.170	35.810	23.970		
SCGF-β [ng/mL]	Mean	191.594	216.834	36.824	<0.0001	1.0000
	SEM	23.854	26.456	1.345		
	Median	197.690	189.664	37.639		
	IQR	176.292	75.318	8.147		
SDF-1α+β [pg/mL]	Mean	1008.943	868.606	1596.160	<0.0001	1.0000
	SEM	84.795	59.952	56.487		
	Median	883.690	831.120	1556.050		
	IQR	305.530	281.040	509.760		
TNF-α [pg/mL]	Mean	59.754	67.218	48.602	0.0026	1.0000
	SEM	3.705	6.845	2.589		
	Median	61.030	64.010	45.940		
	IQR	25.030	29.810	6.200		
TNF-β [pg/mL]	Mean	116.192	61.449	1207.996	<0.0001	1.0000
	SEM	20.697	5.545	10.790		
	Median	63.030	59.440	1209.405		
	IQR	44.050	28.350	65.120		
TRAIL [pg/mL]	Mean	38.398	44.284	19.951	<0.0001	1.0000
	SEM	3.791	4.316	1.215		
	Median	34.180	41.890	18.380		
	IQR	16.800	12.710	5.710		
VEGF [pg/mL]	Mean	258.338	258.141	18.010	<0.0001	1.0000
	SEM	44.980	37.547	0.000		
	Median	231.320	249.770	18.010		
	IQR	353.430	199.070	0.000		

Abbreviations used: CTACK: cutaneous T-cell-attracting chemokine; Eotaxin: eosinophil chemotactic protein; Basic FGF: basic fibroblast growth factor; G-CSF: granulocyte colony-stimulating factor; GM-CSF: granulocyte-macrophage colony-stimulating factor; GRO-α: growth-regulated alpha protein; HGF: hepatocyte growth factor; IFN-α2: interferon alpha-2; IFN-γ: interferon gamma; IL-1α: interleukin 1 alpha; IL-1β: interleukin 1 beta; IL-1ra: interleukin 1 receptor antagonist; IL-2: interleukin 2; IL-2Rα: interleukin 2 receptor alpha; IL-3: interleukin 3; IL-4: interleukin 4; IL-5: interleukin 5; IL-6: interleukin 6; IL-7: interleukin 7; IL-8: interleukin 8; IL-9: interleukin 9; IL-10: interleukin 10; IL-12 (p70): interleukin 12 (p70); IL-12 (p40): interleukin 12 (p40); IL-13: interleukin 13; IL-15: interleukin 15; IL-16: interleukin 16; IL-17A: interleukin 17A; IL-18: interleukin 18; IP-10: interferon gamma-induced protein 10; IQR: interquartile range; LIF: leukemia inhibitory factor; MCP-1: monocyte chemoattractant protein-1; MCP-3: monocyte chemoattractant protein-3; M-CSF: macrophage colony-stimulating factor; MIF: macrophage migration inhibitory factor; MIG: monokine induced by gamma interferon; MIP-1α: macrophage inflammatory protein 1 alpha; MIP-1β: macrophage inflammatory protein 1 beta; β-NGF: beta-nerve growth factor; PDGF-BB: platelet-derived growth factor BB; RANTES: regulated on activation, normal T cell expressed and secreted; SCF: stem cell factor; SCGF-β: stem cell growth factor beta; SDF-1α+β: stromal cell-derived factor 1 alpha and beta; SEM: standard error of mean; TNF-α: tumor necrosis factor alpha; TNF-β: tumor necrosis factor beta; TRAIL: TNF-related apoptosis-inducing ligand; and VEGF: vascular endothelial growth factor. The *p*-values listed in the table are the result of either one-way ANOVA or the Kruskal–Wallis test comparing all groups within the study: smoking HNC patients, non-smoking HNC patients, and healthy controls.

**Table 3:** Results of post hoc analysis, with statistical significance determined at *p* < 0.05.

Parameter	*p*-Value		
	Smoking HNC vs. Non-Smoking HNC	Smoking HNC vs. Control	Non-Smoking HNC vs. Control
CTACK	0.3244	0.0003	<0.0001
IL-7	0.9650	<0.0001	<0.0001
Basic FGF	1.0000	0.0006	<0.0001
G-CSF	0.3339	<0.0001	<0.0001
GM-CSF	1.0000	0.0347	0.0347
HGF	0.1570	<0.0001	<0.0001
IFN-α2	1.0000	<0.0001	<0.0001
IFN-γ	0.1565	0.0038	<0.0001
IL-1α	1.0000	<0.0001	<0.0001
IL-1β	1.0000	<0.0001	<0.0001
IL-1ra	1.0000	<0.0001	<0.0001
IL-2	0.7074	0.0029	0.0001
IL-2Rα	0.1998	<0.0001	<0.0001
IL-3	0.1059	<0.0001	<0.0001
IL-6	0.5234	<0.0001	<0.0001
IL-8	1.0000	0.0010	0.0149
IL-9	0.3684	<0.0001	<0.0001
IL-10	0.2873	0.3726	0.0069
IL-12 (p70)	0.9328	0.0002	0.0029
IL-12 (p40)	1.0000	<0.0001	<0.0001
IL-13	1.0000	0.0004	<0.0001
IL-15	1.0000	0.0765	0.0347
IL-17A	1.0000	<0.0001	<0.0001
IL-18	0.1308	0.0001	<0.0001
IP-10	0.3760	0.0021	<0.0001
LIF	1.0000	0.0025	0.0021
MCP-1	1.0000	0.0009	<0.0001
MCP-3	0.6000	<0.0001	<0.0001
M-CSF	0.0484	<0.0001	<0.0001
MIF	0.6647	0.0011	<0.0001
MIG	0.5514	<0.0001	<0.0001
MIP-1α	0.3406	<0.0001	<0.0001
MIP-1β	0.4054	<0.0001	<0.0001
PDGF-BB	1.0000	0.0362	0.1105
RANTES	1.0000	<0.0001	<0.0001
SCF	0.6643	0.0480	0.0009
SCGF-β	1.0000	<0.0001	<0.0001
SDF-1α+β	1.0000	<0.0001	<0.0001
TNF-α	1.0000	0.0224	0.0053
TNF-β	0.2971	<0.0001	<0.0001
TRAIL	0.4868	<0.0001	<0.0001
VEGF	1.0000	<0.0001	<0.0001

Abbreviations used: CTACK: cutaneous T-cell-attracting chemokine; Basic FGF: basic fibroblast growth factor; G-CSF: granulocyte colony-stimulating factor; GM-CSF: granulocyte-macrophage colony-stimulating factor; HGF: hepatocyte growth factor; HNC—head and neck cancer; IFN-α2: interferon alpha-2; IFN-γ: interferon gamma; IL-1α: interleukin 1 alpha; IL-1β: interleukin 1 beta; IL-1ra: interleukin 1 receptor antagonist; IL-2: interleukin 2; IL-2Rα: interleukin 2 receptor alpha; IL-3: interleukin 3; IL-6: interleukin 6; IL-7: interleukin 7; IL-8: interleukin 8; IL-9: interleukin 9; IL-10: interleukin 10; IL-12 (p70): interleukin 12 (p70); IL-12 (p40): interleukin 12 (p40); IL-13: interleukin 13; IL-15: interleukin 15; IL-17A: interleukin 17A; IL-18: interleukin 18; IP-10: interferon gamma-induced protein 10; LIF: leukemia inhibitory factor; MCP-1: monocyte chemoattractant protein-1; MCP-3: monocyte chemoattractant protein-3; M-CSF: macrophage colony-stimulating factor; MIF: macrophage migration inhibitory factor; MIG: monokine induced by gamma interferon; MIP-1α: macrophage inflammatory protein 1 alpha; MIP-1β: macrophage inflammatory protein 1 beta; PDGF-BB: platelet-derived growth factor BB; RANTES: regulated on activation, normal T cell expressed and secreted; SCF: stem cell factor; SCGF-β: stem cell growth factor beta; SDF-1α+β: stromal cell-derived factor 1 alpha and beta; TNF-α: tumor necrosis factor alpha; TNF-β: tumor necrosis factor beta; TRAIL: TNF-related apoptosis-inducing ligand; and VEGF: vascular endothelial growth factor.

## Data Availability

The data presented in this study are available upon request from the corresponding author. The data are not publicly available due to privacy/ethical restrictions.

## Supplementary Materials

The following supporting information can be downloaded at https://www.mdpi.com/article/10.3390/biomedicines12040748/s1: Figure S1: Spearman’s rank correlation analysis.
