# Supplementary figures and images for: Exploring the Link between Inflammatory Biomarkers and Head and Neck Cancer: Understanding the Impact of Smoking as a Cancer-Predisposing Factor
Source: Biomedicines. 2024 Mar 27;12(4):748. doi: 10.3390/biomedicines12040748 (PMC11048483; doi:10.3390/biomedicines12040748)

Head and neck cancer (HNC) smoking group

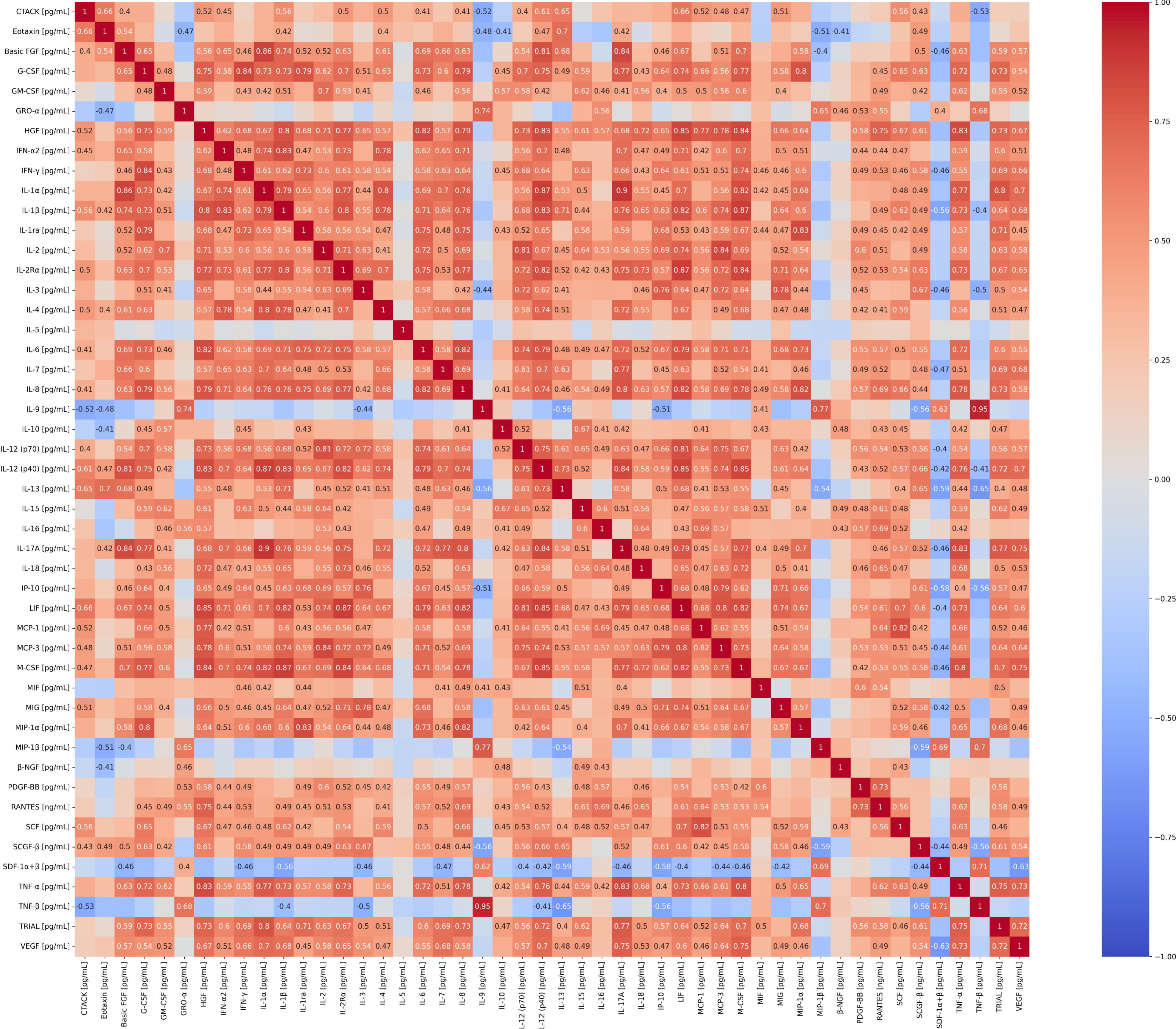

Head and neck cancer (HNC) non-smoking group

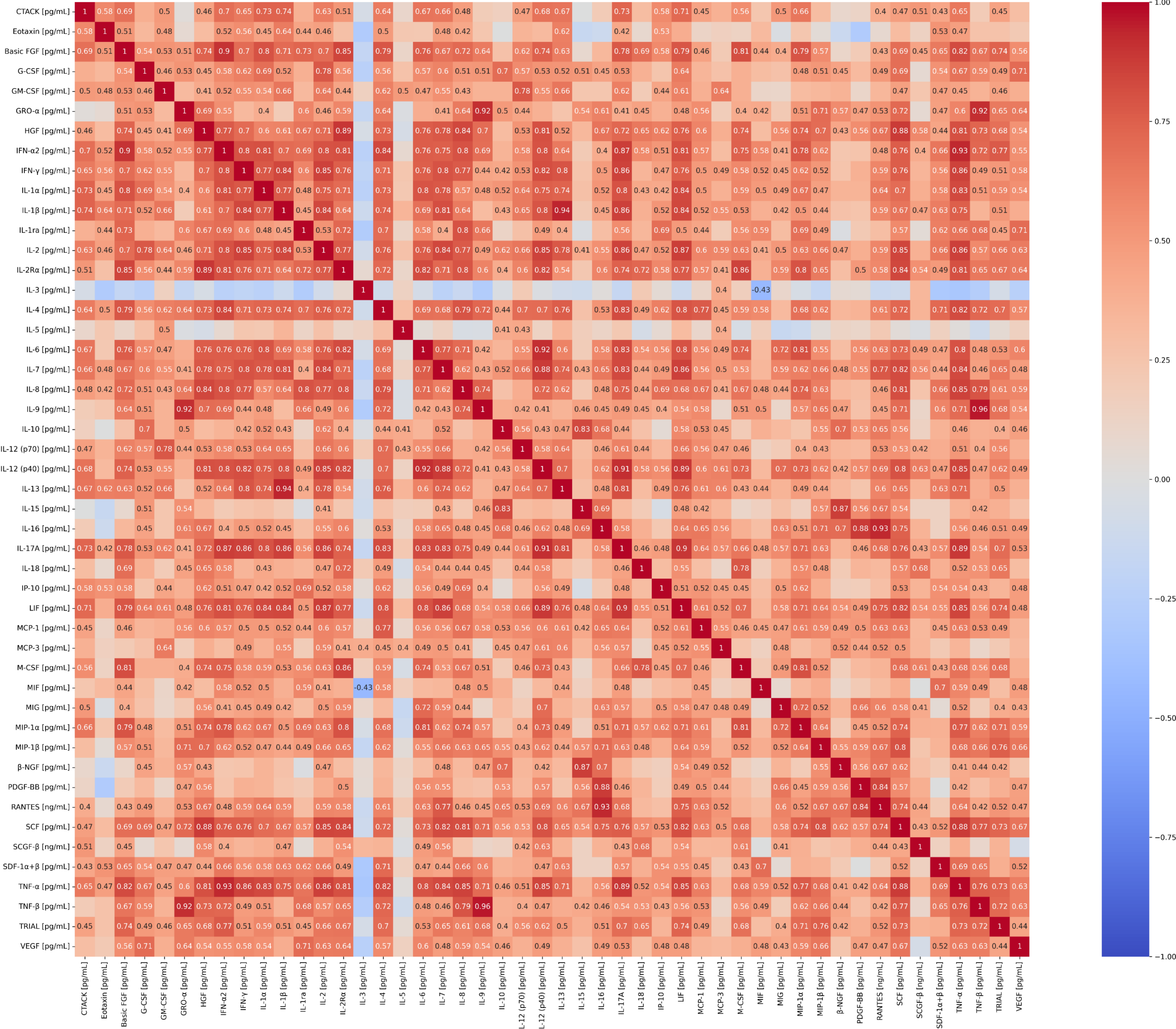

Control group

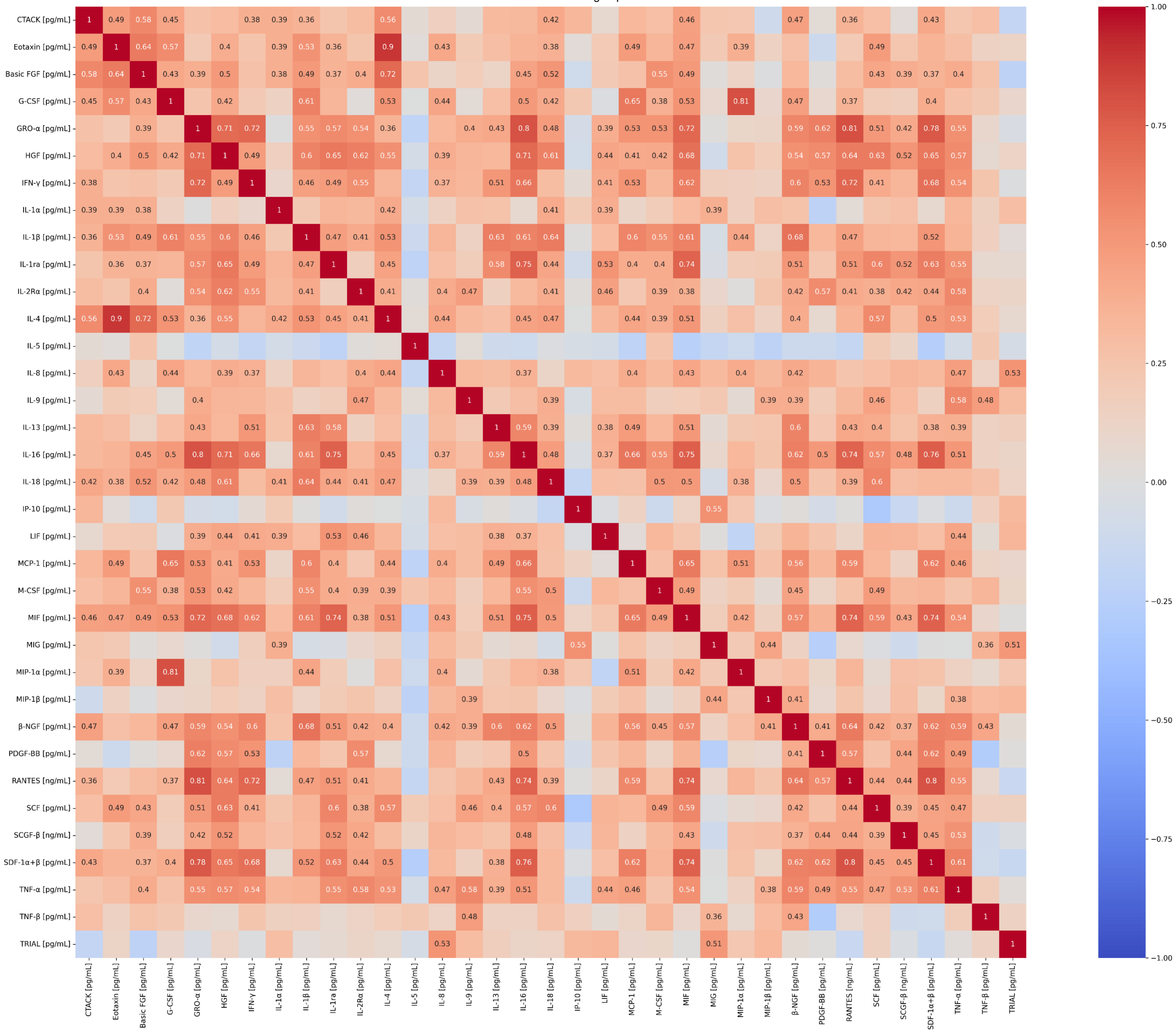

Supplement: Supplementary file 1 [file biomedicines-12-00748-s001.zip › biomedicines-2919118-supplementary.pdf]
